# The universal existence of myodural bridge in mammals: an indication of a necessary function

**DOI:** 10.1038/s41598-017-06863-z

**Published:** 2017-08-15

**Authors:** Nan Zheng, Xiao-Ying Yuan, Yan-Yan Chi, Pei Liu, Bing Wang, Jia-Ying Sui, Seung-Ho Han, Sheng-Bo Yu, Hong-Jin Sui

**Affiliations:** 1 Department of Anatomy, College of Basic Medicine, Dalian Medical University, Dalian, P.R. China; 2 Dalian Hoffen Preservation Technique Institution, Dalian, P.R. China; 3 Department of Anatomy, College of Medicine, Chung-Ang University, Seoul, Korea

## Abstract

The “myodural bridge” was described in literatures as a dense fibrous tissue connecting the sub-occipital musculature with the spinal dura mater in human studies. Now the concept of “myodural bridge” was perceived as an exact anatomical structure presumably essential for critical physiological functions in human body, and might exist in other mammals as well. To determine the existence of the “myodural bridge” in other mammals and to lay a foundation for the functional study, we examined representatives in five different mammalian orders. Based on the anatomical dissections, P45 plastinated sections and histological sections, we found that a dense fibrous tissue connected the rectus capitisdorsalis minor and the spinal dura mater through the dorsal atlanto-occipital interspace with or without the medium of the posterior atlanto-occipital membrane. These observed connective tissues were very similar to the “myodural bridge” previously described in humans. We proposed that the “myodural bridge”, as an evolutionally conserved structure, presents in many other mammals. Moreover, we believed that the “myodural bridge” might be a homologous organ in mammals. Thus, this study could provide an insight for our understanding the physiological significance of the “myodural bridge”, especially in human.

## Introduction

The sub-occipital region is one of the most complex anatomical regions in the human body. Many studies were focus on the sub-occipital muscles in this region. In 1995, a connective tissue link was described in humans between the rectus capitis posterior minor (RCPmi) and the cervical spinal dura mater. Dissections of 11 human cadavers by Hack *et al*.^1^ revealed a dense band of tissue connecting the RCPmi and the posterior atlanto-occipital (PAO) membrane. They also found that the PAO membrane was connected to the spinal dura mater by many fine connective tissue bands. So they termed this tissue connection as the “myodural bridge”(MDB)^1^. Subsequent gross dissections, histological, and imaging studies showed that this connective tissue also exists between the rectus capitis posterior major (RCPma), the oblique capitis inferior (OCI), the nuchal ligament and the spinal dura mater through the posterior atlanto-axial interspace in humans^2–11^.

A variety of functions of the MDB were speculated in last two decades. It was suggested that the MDB might act to prevent in-folding of the spinal dura mater during head extension^1^, or act to trigger cervical neck extensors that would resist hyperflexion or hypertranslation^12^, or play a role in maintaining the integrity of the subarachnoid space^5^, or work as a pump to propel the circulation of cerebral spinal fluid^13^. But those proposed functions have not been demonstrated so far. Now that the MDB was an exact anatomical structure with potential important physiological functions in human body, it might widely present in mammals and play a corresponding role as well. To determine the universal existence of the MDB in mammals and to lay the foundation for exploring the MDB’ functions in humans, we examined the presence of the MDB in seven different mammals with a combination of techniques including gross dissection, P45 sheet plastination and histological staining.

## Results

### The formation of the post-occipital muscles was determined based upon the gross dissection

**Figure 1:**
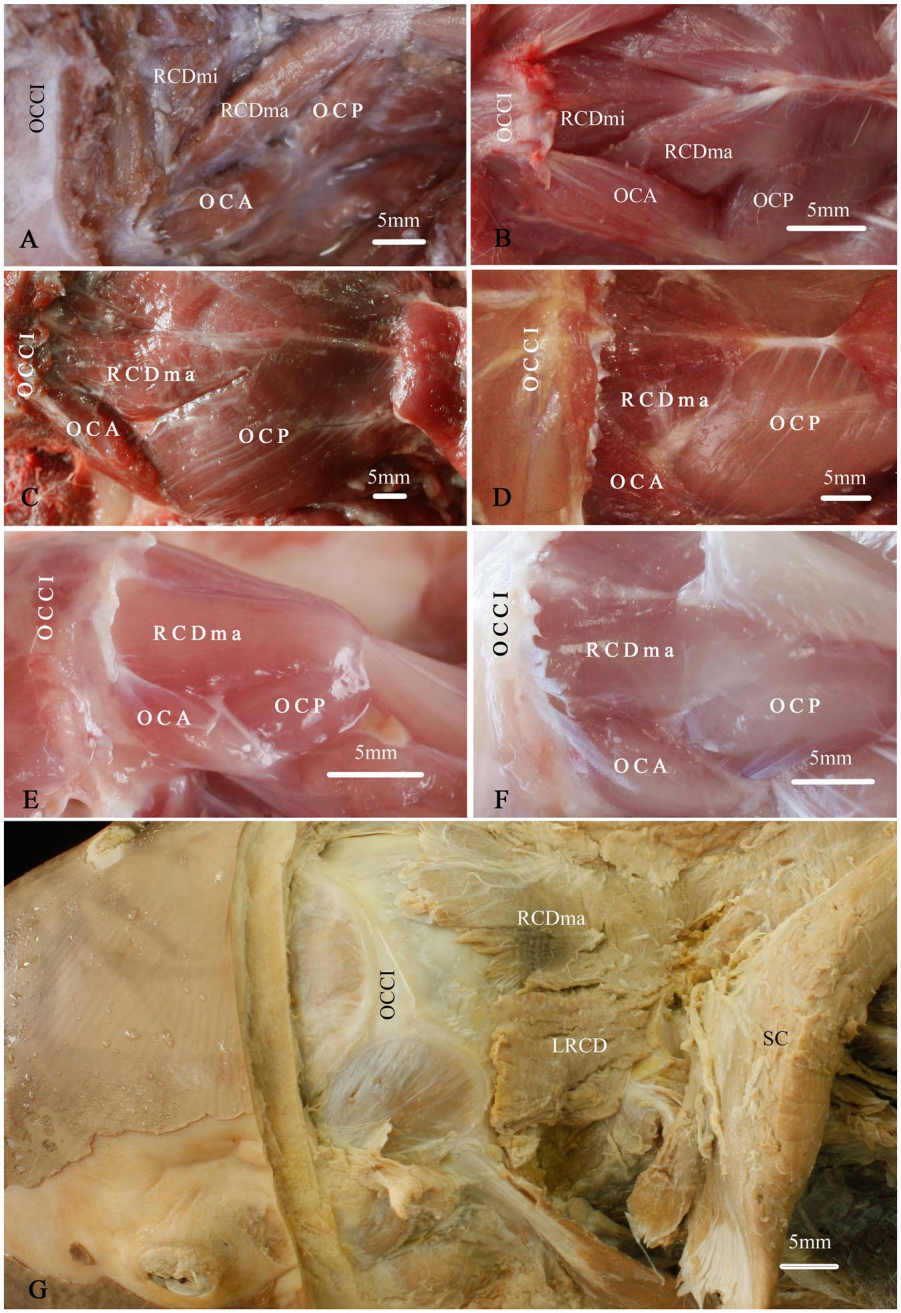
The post-occipital muscle group of five mammalian orders of mammals showed by the gross dissection (dorsal and lateral view). (**A**) *Macacamulatta*. (**B**) *Oryctolaguscuniculus*. (**C**) *Canisfamiliaris*. (**D**) *Feliscatus*. (**E**) *Ratusnorvegicus*. (**F**) *Caviaporcellus*. (**G**) *Indoasian finless porpoise*. The post-occipital muscles of *Macacamulatta*, *Oryctolaguscuniculus*, *Canisfamiliaris*, *Feliscatus*, *Ratusnorvegicus*, *Caviaporcellus* were composed of the rectus capitis dorsal major (RCDma), rectus capitis dorsal minor (RCDmi), oblique capitis anterior (OCA), and oblique capitis posterior (OCP). The RCDmi was covered almost entirely by the RCDma in *Canisfamiliaris*, *Feliscatus*, *Ratusnorvegicus*, and *Caviaporcellus* (**C**,**D**,**E**,**F**). While in *Indoasian finless porpoise*, the post-occipital muscles were composed of the rectus capitis dorsal minor (RCDmi), rectus capitis dorsal major (RCDma) and lateral rectus capitis dorsal (LRCD). The RCDma and LRCD were found in parallel arranged in the post-occipital region and the RCDmi was just ventral to the RCDma. SC: the semispinaliscapitis. OCCI: the occipital bone.

**Figure 2:**
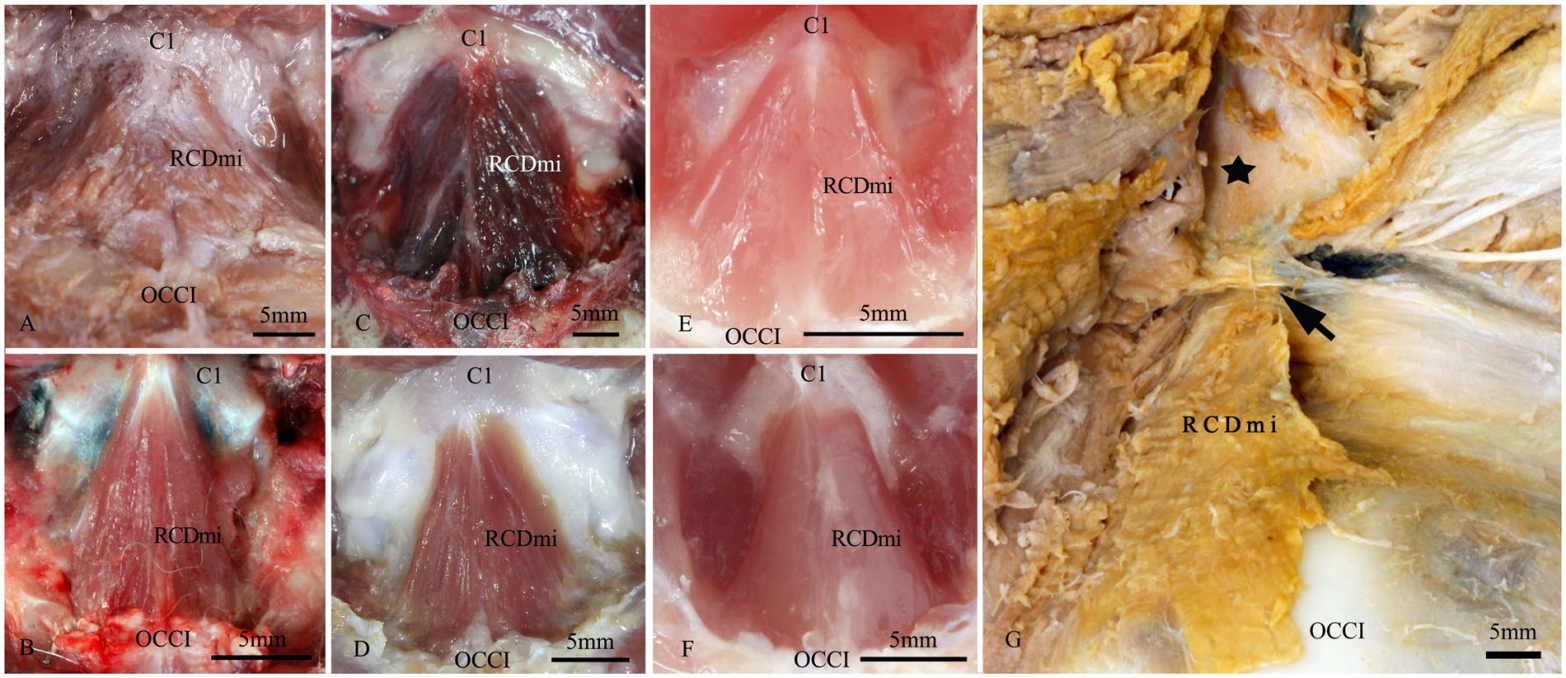
The RCDmi of the five mammalian orders of mammals showed when the RCDma removed (antero-superior view). (**A**) *Macacamulatta*. (**B**) *Oryctolaguscuniculus*. (**C**) *Canisfamiliaris*. (**D**) *Feliscatus*. (**E**) *Ratusnorvegicus*. (**F**) *Caviaporcellus*. (**G**) *Indoasian finless porpoise*. The RCDmi was originated from the dorsal arch of the atlas (C1) and inserted into the occipital bone (OCCI) in *Macacamulatta*, *Oryctolaguscuniculus*, *Canisfamiliaris*, *Feliscatus*, *Ratusnorvegicus*, *Caviaporcellus*. While the RCDmi of *Indoasian finless porpoise* was originated from the occipital bone (OCCI) and extended into the posterior atlanto-occipital interspace (↑). ★: The fused spinous process. RCDmi: the rectus capitis dorsal minor.

In the post-occipital (same as sub-occipital in humans)region of five mammalian orders of mammals, the post-occipital muscle group was found locating dorsally to the atlas (C1) and the axis (C2), and originating from or inserting into the occipital bone, the C1, or the C2 (Figs 1 and 2). In *Macacamulatta, Canisfamiliaris, Feliscatus, Oryctolaguscuniculus, Rattusnorvegicus and Caviaporcellus*, the post-occipital muscles were found to consist of the rectus capitis dorsal major (RCDma, same as RCPma in humans), the rectus capitis dorsal minor (RCDmi, same as RCPmi as in humans), the oblique capitis anterior (OCA, same as OCS in humans), and the oblique capitis posterior (OCP, same as OCI in humans) (Fig. 1A–F). Among them, the RCDma and RCDmi were located along the posterior median line. The RCDma was originated from the spinous process of the C2 and inserted into the occipital bone. Medially and ventrally to the RCDma, the RCDmi was originated from the dorsal arch of the C1 and inserted into the occipital bone (Fig. 2A–F). The OCA and OCP were laterally to the RCDma and RCDmi. The former extended from the occipital bone to the transverse process of C1 and the latter from the transverse process to the spinous process of the C2. Different from that of the above-mentioned mammals, the post-occipital muscles of *Indoasian finless porpoise*, were found to be composed of RCDma, RCDmi and the lateral rectus capitis dorsal (LRCD) (Figs 1G and 2G). The RCDma and LRCD were extended from the fused spinous process of first three cervical vertebrae to the occipital bone. These two muscles were nearly parallel in arrangement in the post-occipital region of the *Indoasian finless porpoise* (Fig. 1G). While the RCDmi was originated from the occipital bone and extended into the posterior atlanto-occipital interspace under the RCDma (Fig. 2G). In addition, no oblique muscles were found in the post-occipital muscle group of the *Indoasian finless porpoise*.

As a result, the RCDmi presents in the five different kind of mammalian orders co*nfi*rmed by its origin and insertion (Fig. 2).

### The connections between the RCDmi and the spinal dura mater with or without intermediary of the PAO membrane were determined based upon the gross dissection

**Figure 3:**
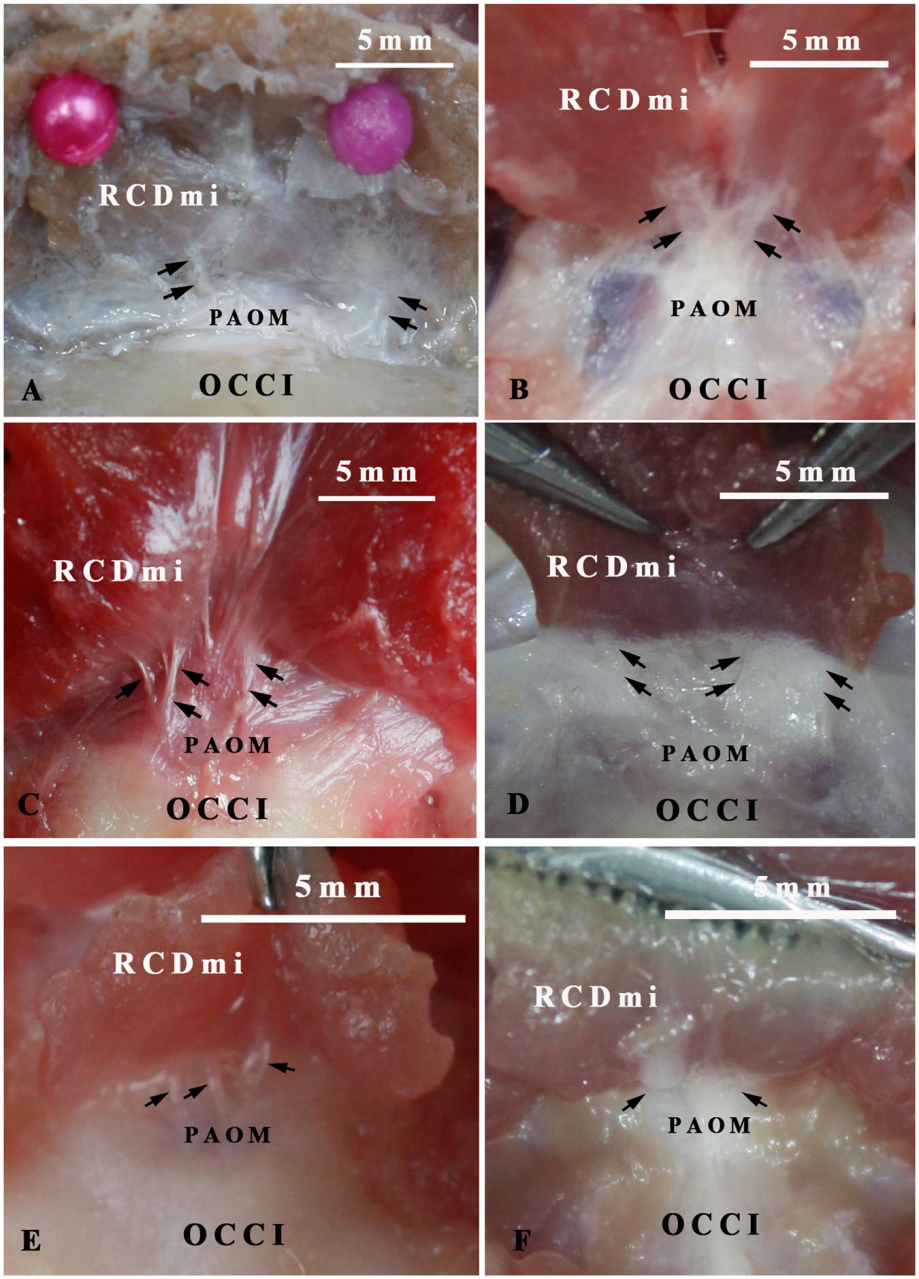
Gross dissection specimens showing the connection between the RCDmi and the PAO membrane (antero-superior view). The rectus capitis dorsal minor (RCDmi) was isolated from the occipital bone and reflected dorsally and caudally. Multi-fibers bundles (→) were discovered protruding from the ventral part of the RCDmi and connecting with the posterior atlanto-occipital membrane (PAOM) in *Macacamulatta* (**A**), *Oryctolaguscuniculus* (**B**), *Canisfamiliaris* (**C**), *Feliscatus* (**D**), *Ratusnorvegicus* (**E**), *Caviaporcellus* (**F**). OCCI: occipital bone.

**Figure 4:**
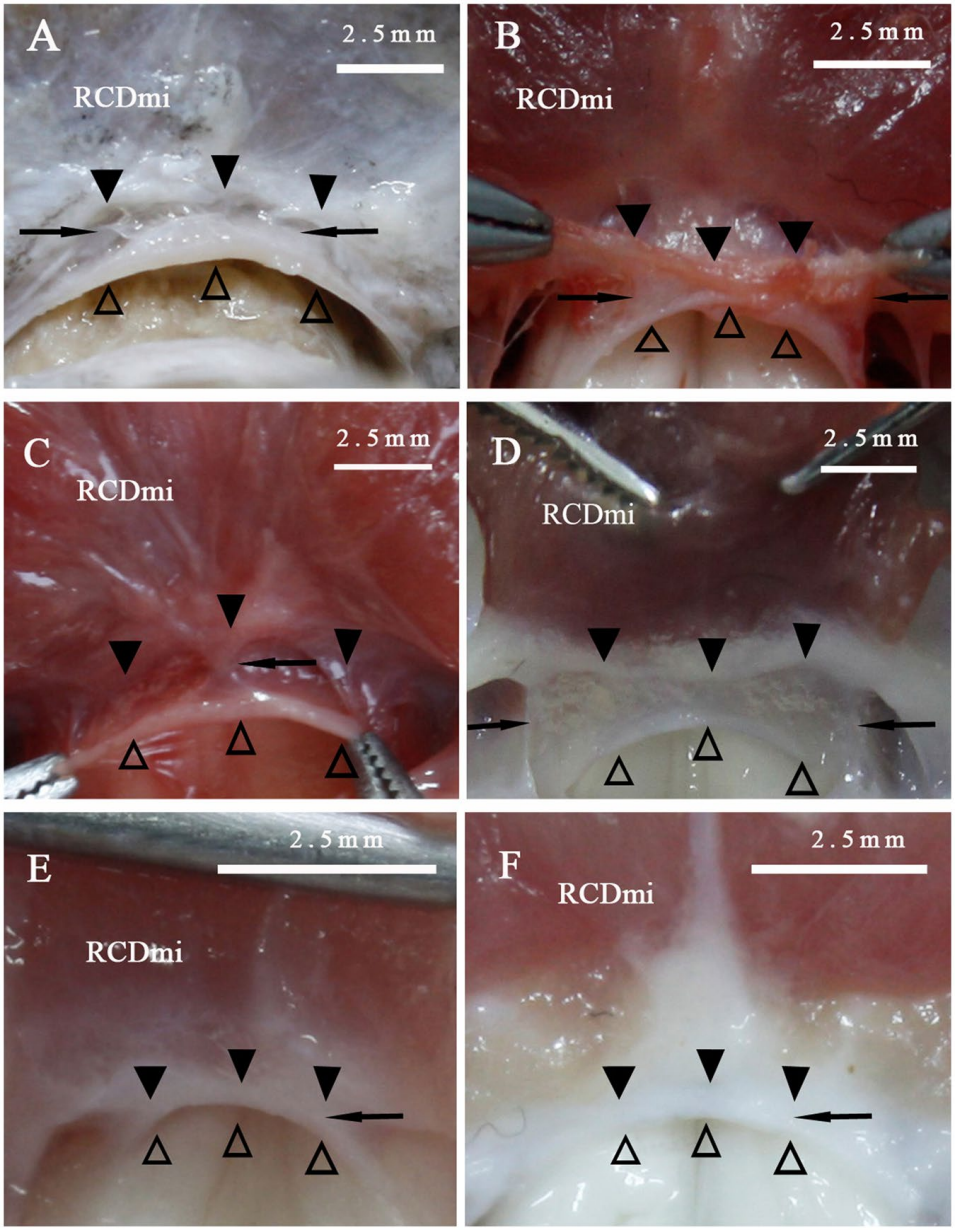
The connections between the PAO membrane and the spinal dura mater (antero-superior view). The PAO membrane and the spinal dura mater were sectioned transversally and simultaneously in *Macacamulatta* (**A**), *Oryctolaguscuniculus* (**B**), *Canisfamiliaris* (**C**), *Feliscatus* (**D**), *Ratusnorvegicus* (**E**), *Caviaporcellus* (**F**). The PAO membrane (▲) and the spinal dura mater (△) were found tightly connected by some dense connective tissues (→) in the dorsal central part of the posterior atlanto-occipital interspace. RCDmi: the rectus capitis dorsal minor.

**Figure 5:**
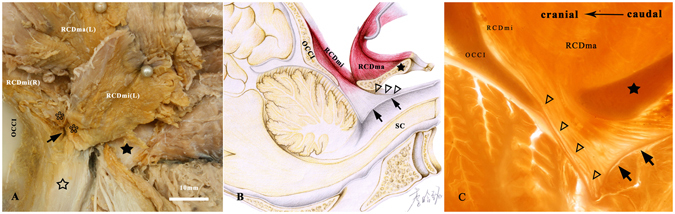
The RCDmi entered the epidural space through the posterior atlanto-occipital interspace and inserted into the spinal dura mater in *Indoasian finless porpoise*. (**A**) Gross dissection specimen of the post-occitiptal region. (**B**) Diagram of the connection of the RCDmi with the dura mater. (**C**) P45 plastinated sheets (median sagittal section) showing the connections between the RCDmi and the spinal dura mater. The muscular bundles (*) of the ventral end of the RCDmi pierced into the posterior atlanto-occipital interspace (↑) and then continued with the tendon-like fibers (Δ) and finally inserted into the dura mater (↑↑). ☆: The capsule of atlanto-occipital joint. OCCI: occipital bone. SC: spinal cord. RCDmi: the rectus capitis dorsal minor. RCDma: the rectus capitis dorsal major. RCDmi (R): the rectus capitis dorsal minor in right side maintained *in situ*. RCDmi (L): the rectus capitis dorsal minor in left side was reflected from the occipital bone. RCDma (L): the rectus capitis dorsal major in left side was reflected from the occipital bone. ★: The fused spinous process. Han-Ming Li at Dalian Medical University was appreciated for her drawing the diagram of Indoasian finless porpoise (Fig. 5B).

In *Macacamulatta*, *Oryctolaguscuniculus*, *Canisfamiliaris*, *Feliscatus*, *Ratusnorvegicus*, *Caviaporcellus*, multiple dense fibrous bundles were discovered to connect the RCDmi with the PAO membrane (Fig. 3). And the PAO membrane was found to tightly adhere to the posterior wall of the cervical spinal dura sac with some connective tissue (Fig. 4). As a result, the MDB was formed by complex connections between the RCDmi and the spinal dura mater. However, in *Indoasian finless porpoise*, the RCDmi was found inserting into the posterior atlanto-occipital interspace directly and no PAO membrane appearing in this interspace (Fig. 5A,B).

### The connections between the RCDmi and the spinal dura mater with or without intermediary of the PAO membrane determined based upon the P45 plastinated sheets of the head and neck

**Figure 6:**
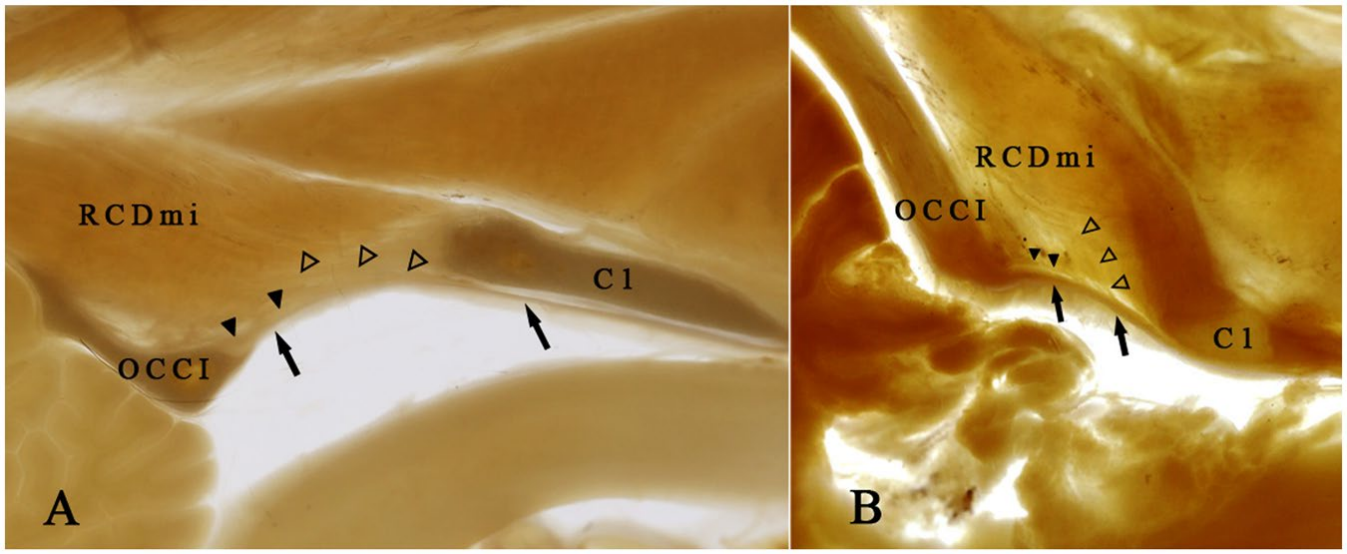
P45 plastinated sheets (median sagittal section) showing the fibrous fusing connections of the RCDmi, the PAO membrane and the spinal dura mater. (**A**) The median sagittal plane of *Canisfamiliaris*. (**B**) The median sagittal plane of *Macacamulatta*. (**A,B**) Showed that the fiber bundles (Δ) were found protruded from the RCDmi and pierced into the PAO membrane (▲), and finally connected with the spinal dura mater (↑). OCCI: occipital bone. C1: atlas. RCDmi: rectus captious dorsal minor.

In the sagittal sections of head and neck of *Macacamulatta* and *Canisfamiliaris*, the RCDmi was showed extending from the posterior arch of the atlas to the occipital bone. The P45 plastinated sheet of *Canisfamiliaris*and *Macacamulatta* shows that a bundle of fibers of RCDmi pierce into the PAO membrane, and finally connect with the spinal dura mater (Fig. 6A,B). While, in *Indoasian finless porpoise*, the RCDmi was found to originate from the occipital bone and run ventrally through the posterior atlanto-occipital interspace then insert into the spinal dura mater directly and no PAO membrane appears at above-mentioned interspaces (Fig. 5B,C).

### Dense fibrous connections between the RCDmi and the spinal dura mater were showed by HE staining

**Figure 7:**
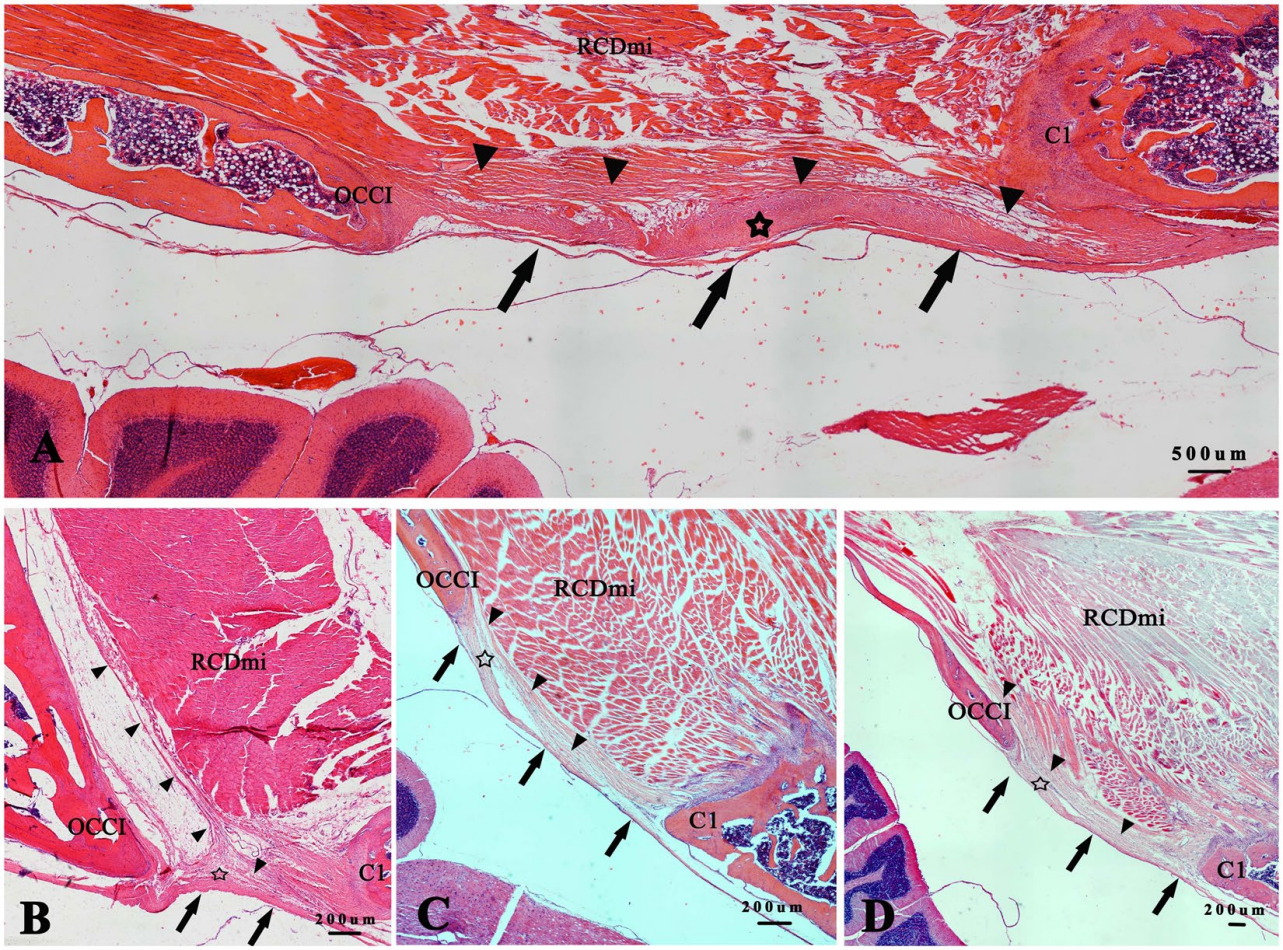
HE stained slides showing the dense fibrous connections between the RCDmi and the spinal dura mater. The dense fibrous bundles from the ventral part of the RCDmi (▲) tightly connected with the PAO membrane in ventral (☆) or passed through the PAO membrane and then fused with the spinal dura mater (↑) in *Oryctolaguscuniculus* (**A**), *Feliscatus* (**B**), *Ratusnorvegicus* (**C**) and*Caviaporcellus* (**D**). The PAO membrane fused with the spinal dura mater tightly in all the slices.

In the sagittal section of the post-occipital region of *Feliscatus*, *Oryctolaguscuniculus*, *Ratusnorvegicus* and *Caviaporcellus*, the ventral muscular fibers of the RCDmi were discovered to give off dense fibrous bundles and some of them connect with the PAO membrane and others pass through the PAO membrane and insert into the spinal dura mater (Fig. 7). Consequently integration of the RCDmi, the PAO membrane and the spinal dura mater was formed via the dense fibrous connections, logically termed as the MDB.

## Discussion

The sub-occipital region is one of the most complex anatomical regions in the human body. The MDB was described in lots of literatures as a dense fibrous tissue connecting the sub-occipital musculature with the spinal dura mater through the posterior atlanto-occipital and atlanto-axial interspaces in this region. In 1995, Hack revealed a dense band of tissue connecting the RCPmi and PAO membrane, and then the latter was intimately attached to the outer surface of the dura mater by a fine connective tissue bridge, especially near the midline^1^. Thus, the connection between the RCPmi and PAO membrane eventually connected with the dura mater was referred as the MDB by Hack *et al*.^1^. Subsequent investigations showed that this connective tissue also existed between the RCPma, OCI, nuchal ligament and the spinal dura mater^2–12^.

As the MDB was found as a normal anatomical structure in human being, it still remained unknown whether this structure universally present in other mammals., In this study, representatives of mammalian orders were examined, including tree-dwelling primates (*Macacamulatta*), land-based carnivores (*Canisfamiliaris* and *Feliscatus*), Cave-dwelling lagomorpha (*Oryctolaguscuniculus*) and rodentia (*Ratusnorvegicus* and *Caviaporcellus*), and aquatic cetaceans (*Indoasian finless porpoise*). Firstly, we found that the post-occipital muscle group consistently appears in the post-occipital region of these mammals. It consists of the RCDma, RCDmi, OCA and OCP in *Macacamulatta*, *Canisfamiliaris*, *Feliscatus*, *Oryctolaguscuniculus*, *Ratusnorvegicus* and *Caviaporcellus*, which are in agreement with previous results^14–16^. And slightly different from above-mentioned mammals, in *Indoasian finless porpoise*, the post-occipital muscle group is composed of the RCDma, RCDmi, and LRCD. This might be due to the fusion of its first three cervical vertebrae, which could make its body more suitable for underwater movement^17, 18^. Among the post-occipital muscles, the RCDmi was observed intimately adjacent to the posterior atlanto-occipital interspace and extending from the posterior arch of the atlas to the occipital bone in *Macacamulatta*, *Canisfamiliaris, Feliscatus, Oryctolaguscuniculus, Ratusnorvegicus*and *Caviaporcellus*, while in *Indoasian finless porpoise*, the RCDmi was found extending from the occipital bone into the posterior atlanto-occipital interspace. As a whole, the formation of the post-occipital muscles of these mammals was similar to that of the sub-occipital muscles of human being.

Based on the results of gross dissections and P45 plastinated sheets and HE staining of histological sections, our study shows that the integration of the RCDmi, the PAO membrane and the posterior wall of the cervical spinal dura sac is formed via multiple dense fibrous bundles, which originate from the ventral aspect of the RCDmi and fuse with the PAO membrane and some of them pass through the PAO membrane and insert into the spinal dura mater in *Macacamulatta*, *Canisfamiliaris, Feliscatus, Oryctolaguscuniculus, Ratusnorvegicus and Caviaporcellus*. As a consequence, the existence of the MDB will be determined in these mammals according to the anatomical features of MDB defined in the human being^1^. In *Indoasian finless porpoise*, the MDB was a kind of direct connection because the RCDmi directly enters into the epidural space through the posterior atlanto-occpitial interspace and then inserts into the spinal dura mater without the PAO membrane relaying. The MDB strengthened and occupied the opened posterior atlanto-occpitial interspace in *Indoasian finless porpoise* might be a result of a limited movements of head and neck caused by the fusion of the first three cervical vertebrae. Thus we concluded that the MDB present in the mammals of the five mammalian orders observed in our study, although they live in different environments. Although different from upright walk style of human^19^, the MDB appears in a marine mammal (*Indoasian finless porpoise*) and terrestrial quadrupeds (*Macacamulatta*, *Canisfamiliaris*, *Feliscatus*, *Oryctolaguscuniculus*, *Ratusnorvegicus* and *Caviaporcellus*) in the deepest layer of the napex. And, consequently, it was speculated that the MDB would be a universal existing, normal anatomical structure in mammals.

The morphological structural changes are thought to be functional adaptations^20, 21^. Now we provide anatomical evidence to support the notion that the MDB found in humans^1–12^ also presents in multiple mammals in our study, its function might be important and necessary for both human and other mammals. Previous studies suggested that the “myodural bridge” would fulfill several major functions, such as preventing in-folding of the dura mater during head extension^1^, which may act to trigger cervical neck extensors and resist hyperflexion or hypertranslation^12^. Although intensive studies were made by many researchers, the function of the MDB is still unclear. Recently, Sui *et al*.^13, 22^ hypothesized that the MDB might have an effect on the circulation of cerebral spinal fluid (CSF). And this hypothesis was validated by the cerebrospinal fluid circulation experiment further^13^. This study showed that the integration of the RCDmi, PAO membrane and dorsal wall of the dura sac connected by the MDB could maintain the integrity of the subarachnoid space caudal to the cerebellomedullary cistern, which is in agreement with the previous studies by Scali *et al*.^5, 23^. However, under dynamic considerations, during the movements of head and neck, the RCDmi or RCPmi could more efficiently pull the dura sac along the MDB to change the pressure and volume of the sac, thus propelling the CSF circulation at the junction of cranial cavity and vertebral canal, especially the RCDmi in *Indoasian finless porpoise* due to its directly insert into the spinal dura mater through the dorsal atlanto-occipital interspace. In this case, the results of this study were conductive to explain the function of the MDB as a pump to propel the CSF circulation and support Sui’s hypothesis^13, 22^.

The existence and shape of an anatomical structure could always be influenced by its functions. Less useful structures will gradually degenerate during evolution.Now that the MDB is a universal, normal anatomical structure in many mammals, it should have an important physiological function. Moreover, we believed that the MDB is a homologous organ. For the next stage, morphological differences of the MDB among different kinds of mammals will be addressed in order to exploring the effects of ecological characteristics and living habits on it.

## Conclusion

The results of this study reveal that the MDB might be a universal existing normal anatomical structure in mammals and it should play a key role on integration of the sub-occipital muscular system and the central nervous system. The morphological results of this study support the CSF circulation power hypothesis of the MDB and provide a comparative anatomical basis for the functional study on the MDB.

## Materials and Methods

### Ethics statement

All mammals used in this study were collected with the permission of Chinese Authorities for Animal Protection and the approval of the Ethics Committee of Dalian Medical University. All experiments were performed in accordance with the guidelines and regulations of Dalian Medical University.

Four *Indoasian finless porpoise* cadavers were collected from beaches in Dalian. Four *Macacamulatta* specimens were collected from several zoos in Liaoning Province. Six *Canisfamiliaris*, six *Feliscatus*, six *Oryctolaguscuniculus*, six *Ratusnorvegicus* and six *Caviaporcellus* were obtained from animal experimental center of Dalian Medical University. The obtained specimens had been embalmed through the aorta with the solution containing 10% formalin. Photographic documentation was acquired with a Canon D-40 camera.

### Gross dissection

We dissected two*Indoasian finless porpoise*, two *Macacamulatta*, four *Canisfamiliaris*, four *Feliscatus*, four *Oryctolaguscuniculus*, four *Ratusnorvegicus* and four *Caviaporcellu*. The soft tissues were denuded at the level of the superior nuchal line to the seventh cervical vertebra. Then, the post-occipital muscles were exposed, and the origins and insertions of each muscle were identified. The RCDmi was detached from its occipital attachment, and was reflected toward the atlas (C1) to observe the connection between the RCDmi and the PAO membrane. An incision was made in the PAO membrane along the posterior border of the foramen magnum to observe the connection between the PAO membrane and the spinal dura mater.

### P45 plastinated sheets of the head and neck

The heads and necks of two *Indoasian finless porpoise*, two *Canisfamiliaris*, and two *Macacamulatta* specimens were sliced sagittally with a P45 sheet plastination technique^24, 25^.

### Slicing

The embalmed specimens of the head and neck were frozen at −70 °C for two weeks and then embedded in polyurethane foam and kept at −70 °C for another two days. After freezing, 3 mm sagittal slices were made with a high-speed band saw.

### Bleaching

All the slices were rinsed overnight in cold running water, and afterwards, the slices were immersed in 5% dioxogen overnight.

### Dehydration

After bleaching, the slices were dehydrated with 100% acetone by the freeze substitution method^24, 25^.

### Casting and forced impregnation

After dehydration, the casting mold was prepared. The slices were lifted from the acetone bath and placed between two glass plates. The molds were then filled with polyester (Hoffen polyester P45, Dalian Hoffen Bio-Technique Co. Ltd., Dalian, P. R. China).

The filled mold was then placed upright into a vacuum chamber at room temperature for impregnation. The large bubbles on the surface of the slices were removed manually with a 1-mm stainless steel wire. The absolute pressure was slowly decreased to 20, 10, 5, and 0 mm Hg, according to the bubble releasing. The pressure was maintained at 0 mm Hg until bubbling ceased. The impregnation lasted for more than eight hours.

### Curing

After the vacuum was released, the air bubbles within the sheets were checked and removed. The alignment of the slices was checked and corrected using a stainless steel wire. The top of the mold was clamped with large fold back clamps, and the sheet was then ready for curing. The sheets were cured using a heated water bath and were placed upright in a water bath at 40 °C for 3 days.

### Cutting and sanding the molds

After curing, the sheets were removed from the bath and cooled to room temperature in a rack. The slices were then removed from the flat chamber and covered appropriately with adhesive plastic wrap for protection. A mini-band saw was used to cut and trim the plastic along the edges approximately 1 mm outside the slices. Then, a wool sander was used to remove the sharp edges of the slices. After sanding, the adhesive plastic wrap was removed, and the slices were placed in non-adhesive plastic wrap to avoid scratches. The head-neck sheets were then observed and photographed.

#### HE Staining

Two *Feliscatus*, two *Oryctolaguscuniculus*, two *Ratusnorvegicus* and two Caviaporcellus were used for histological study. Tissue samples were obtained including the intact components of the deep part of post-occipital region ranged from the base of occipital bone to the atlas in cranial-caudal direction and from the RCDma to the spinal dura mater in ventral-dorsal direction. The tissue blocks were sectioned with thickness of 12–15 um, and stained with hematoxylin and eosin. Stained sections were photographed with an NIKON research light microscope at 4x magnification. Multiple images from each section were stitched together using microsoft image composite editor of NIKON Eclipse80i computer processing and analysis system.
